# Adaptation and Psychometric Assessment of the Turkish Version of the Perceived Access to Health Care Questionnaire: Validity and Reliability Analysis

**DOI:** 10.3390/healthcare13040370

**Published:** 2025-02-10

**Authors:** Salim Yilmaz, Metin Ateş, Perihan Abay

**Affiliations:** 1 Department of Health Management, Faculty of Health Sciences, Acibadem Mehmet Ali Aydinlar University, Istanbul 34752, Turkey; 2 Department of Health Management, Faculty of Health Sciences, Istanbul Arel University, Istanbul 34537, Turkey; metinates@arel.edu.tr; 3 Kanuni Sultan Süleyman Training and Research Hospital, Istanbul 34303, Turkey; perimsu2006@gmail.com

**Keywords:** access to health services, Turkish health services, perception measurement, reliability analysis, validation study

## Abstract

**Background/Objectives:** Access to health services is a fundamental element of social welfare and individual quality of life. This study aimed to fill gaps in the Turkish literature regarding the assessment and perception of access to health services and to introduce a valid and reliable measurement tool for this purpose. **Methods:** This methodological cross-sectional study was conducted in Istanbul with 639 adults aged 18–65 years. Linguistic and cultural appropriateness were evaluated, and validation was assessed through known group validity using sociodemographic factors. Convergent and divergent validity analyses were performed. Reliability was examined using alpha coefficients and 27% percentile discrimination. A secondary confirmatory factor analysis provided the overall score for the scale. **Results:** The scale was validated on four factors, and seven items were removed during refinement, resulting in a final twenty-three-item scale. Internal consistency was robust, with alpha coefficients of 0.899 for acceptability and affordability, 0.825 for availability, 0.773 for accommodation, 0.892 for awareness, and 0.943 for overall access. Perceived access to health services was significantly correlated with age, beliefs in easy access to health, and satisfaction with outpatient care. **Conclusions:** The validated scale provides a reliable tool for measuring perceptions of access to health services, which is essential for shaping health policies and practices. Comprehensive assessments using such tools can help discern nuanced distinctions between perceived and actual access.

## 1. Introduction

Access to health services is of great importance for the social well-being and quality of life of individuals. This accessibility is measured by each person’s ability to obtain the health services they need and is influenced by various factors, including geographic location, cost, cultural and linguistic barriers, insurance coverage, disparities in healthcare provision, and the identification of individuals with special needs [1,2]. In particular, individuals living in rural areas face various challenges in accessing such services, highlighting the prevalence of barriers in such settings. Furthermore, the common barrier of cost emerged as one of the most important barriers directly impeding individuals’ access to health services [1,3]. The impact of these circumstances extends beyond the individual and has a profound effect on society as a whole [3,4].

According to a 2019 joint report by the World Health Organization (WHO) and the World Bank, 400 million individuals worldwide lack access to basic health services. Most of these individuals are concentrated in low- and middle-income countries [5]. In these countries, 6% of the population suffers extreme poverty because of health-related expenditures. The WHO and the World Bank advocate that all nations should ensure that at least 80% of their population receives primary healthcare. In line with the United Nations’ assertion that health is an inherent human right, the pursuit of universal health coverage is highlighted as essential for achieving the Sustainable Development Goals. However, more than half of the world’s population remains inadequately covered by these services [6,7]. More than 800 million individuals are forced to spend at least 10% of their household budget on basic health services [8]. In regions such as Africa, Latin America, and selected parts of Asia, shortages of basic medical supplies, equipment, and drugs compound the predicament; even in developed and developing countries, public health spending can have harmful consequences, posing a challenge to healthcare access [9].

Since the 1970s, the concept of access to health services has been widely debated and remains a multifaceted issue [10]. Experts have different perspectives, with some defining the concept narrowly in terms of the use of healthcare services, whereas others take a broader perspective that encompasses the comprehensive characteristics of the health system [11]. Health service quality, according to Maxwell’s definition, is evaluated through the dimensions of effectiveness, accessibility, acceptability, equity, efficiency, and safety [12]. Penchansky and Thomas [13] significantly contributed to this discourse by identifying five primary dimensions of access: 1. availability, 2. accessibility, 3. affordability, 4. adequacy, and 5. acceptability [14]. The “availability” dimension measures the quantity of services available to individuals, whereas the “accessibility” dimension considers the spatial and temporal aspects between individuals’ homes and health centers. The remaining three dimensions address nonphysical aspects, including cost, service standards, and cultural considerations [13,14]. In particular, the “availability” dimension refers to the physical availability of resources that can provide healthcare services [15]. Accessibility highlights the distance and time of transportation between the patient and the health center [16]. The “affordability” dimension addresses what service individuals can afford [15]. The “adequacy” dimension refers to the responsiveness of health systems to patient needs, demands, and lifestyles [17]. The “acceptability” dimension assesses how patients value healthcare professionals and resources [18].

These dimensions highlight potential barriers to accessing health services. However, some barriers may be more important than others. For example, economic factors may be the most important barriers to access in some developed countries [19]. Furthermore, the quality and availability of services significantly affect interest in and demand for healthcare [20].

The conceptualization of access to healthcare has advanced significantly with efforts to define its key dimensions; however, comprehensive studies on this topic remain limited in the literature [21]. Evaluations typically rely on data collected by government agencies or large organizations [22]. However, perceived access to health services offers valuable insights into the actual level of access and provides an opportunity to elucidate its reflection in society. Furthermore, the community’s perception of access to health services can affect the frequency and willingness of individuals to use such services. If a family perceives a particular health facility as risky or inadequate, their likelihood of using it will decrease [23]. In contrast, if the community perceives a health facility as high-quality and reliable, the demand for its services will increase. In this context, establishing a positive public image is essential for health organizations to encourage individuals to choose their services [7].

This study holds significant importance as it represents the first attempt in the Turkish literature to adapt and validate the Perceived Access to Healthcare Survey. By addressing a notable gap in the field, the research explores both actual access to health services and the community’s perceived access, offering valuable insights into healthcare accessibility in Turkey. Specifically, the lack of research on perceived access to health services highlights the need for this study, which aims to shed light on the current state of healthcare access and guide improvements in policy and practice.

This study highlights the key dimensions that need to be measured in healthcare access and serves as the first assessment tool in the Turkish literature, focusing on participants’ perceptions of access to healthcare. Evaluating the identified factors (Acceptability, Accessibility, Affordability, and Accommodation) is crucial for understanding the barriers and facilitators of healthcare access from the perspective of participants. It aims to provide a solid foundation for identifying specific challenges within the Turkish healthcare system and addressing them effectively, contributing to the improvement of healthcare access for everyone.

## 2. Materials and Methods

This methodological and cross-sectional study was conducted between 11 December 2022 and 6 November 2023 by surveying outpatients at the Kanuni Sultan Süleyman Training and Research Hospital in Istanbul. This study was conducted according to the Declaration of Helsinki and approved by the Institutional Review Board (or Ethics Committee) of Istanbul Arel University (protocol code E-52857131-050.06.04-258830 and 10 March 2023).

### 2.1. Population and Sample

The sample size was determined based on the established practice of exploratory and confirmatory factor analysis (CFA) in methodological scale development studies in the social sciences. Following the recommendations in the literature, data should be collected for at least 10 times the number of variables observed for each variable (or at least 5 times the number of variables in hard-to-reach contexts). In particular, if the number of variables observed is <20, a minimum of 200 subjects is suggested [24]. Thus, when using a scale comprising 30 items, the collection of data from at least 300 individuals for each item is recommended, according to the ratios suggested in the literature. Although this sample of 300 individuals is adequate for exploratory construct validity, CFA requires an additional 300 individuals from a different sample. To comprehensively cover both types of analyses, a minimum of 600 individuals were targeted, and to account for potentially 10% invalid questionnaires, this study targeted >660 participants. Ultimately, this study successfully enrolled 665 individuals after excluding 26 incomplete and invalid questionnaires, resulting in 639 questionnaires available for analysis.

This study focused on individuals aged 18–65 years to ensure comprehension and consistent participation across the target population. While individuals over 65 were not included because of logistical constraints and potential challenges in completing the survey, this limitation is acknowledged as a potential area for future research to ensure a more comprehensive understanding of healthcare access disparities.

### 2.2. Data Collection Tools

The questionnaire comprised four sections. The first section comprises a demographic information form designed to measure the heterogeneity of participants based on sociodemographic characteristics, including sex, income, education level, and age. After a linguistic review of the “Perceived Access to Health Care Questionnaire,” as discussed in Hoseini-Esfidarjani et al. [7], a pilot application form was introduced. This form includes a 5-point Likert scale for the 30 items in the original scale.

To establish an alternative hypothesis for convergent validity, the Outpatient Satisfaction Scale developed by Kaya and Maimaiti [25] was used. The literature indicates a positive correlation between patient satisfaction and improved access to healthcare services [26]. Higher scores on the scale indicate increased ease and satisfaction with the appointment process for “Appointment”; enhanced perception of examination adequacy and responsiveness to patient needs for “Effective Examination”; improved perceptions of interest, respect, and courtesy from healthcare staff for “Staff Attitude”; increased satisfaction with waiting times for “Waiting Time”; greater overall satisfaction and positive impressions of healthcare services for “Overall Satisfaction”. The scale employs a 5-point Likert-type response format.

To establish an alternative hypothesis for divergent validity, the Healthism Scale, developed by Alfrey et al. [27] and adapted into Turkish by Uğraş et al. [28], was used. The scale evaluates two subdimensions: the critique of individual actions and judgment. Higher scores in the Critique of Individual Actions factor indicate a decreased tendency to critique individual actions and a stronger belief that being healthy is associated with individual efforts and choices. Higher scores in the Judgment factor suggest an increased tendency to accept judgments, such as healthy individuals being superior, more successful, attractive, or disciplined. This 5-point Likert scale indicates that as scores increase, attitudes toward the respective subdimensions become more rigid. Based on the literature review and logical reasoning, both attitudes were considered unrelated to perceived access to healthcare services. Therefore, the scale was included in this study to verify orthogonality and ensure that the observed relationships were free from random correlations.

### 2.3. Linguistic and Cultural Validity Studies

The original nomenclature of the scale was the “Perceived Access to Health Care Questionnaire” and this study was approved on 11 December 2022. During the linguistic/cultural validity phase, two linguistic experts were consulted. One of them was from academia in the relevant field, and the other was a linguist from outside the field. The terminological and semantic assessments of the two translations were compared and consolidated into a single form. In the third step, the experts’ ratings were assessed using intraclass correlation. A team of five individuals reviewed the translations and made revisions based on the experts’ suggestions. After revisions, the translations were back-translated by experts. The compatibility of the translations with the English version was then assessed using intraclass correlation coefficients. A final Turkish translation was then performed for the final version with linguistic appropriateness. A cohort of 55 individuals was selected for the pilot study. The heterogeneity of biopsychosocial demographic characteristics was examined. The primary objective was to assess the comprehensibility of the statements. This was achieved by adapting a form proposed by Byrne et al. [29] in the pilot study, which provided a 5-point Likert-type scale for the 30 items of the original scale (1: not understandable at all to 5: Completely understandable). A total of 55 individuals participated in the linguistic and cultural validation phase. This study aimed to exceed the minimum sample size of 50 recommended in the literature for a pilot study, and 55 individuals were included. Pilot study participants aged between 18 and 65 years (Mean = 31.31, SD = 13.38) were recruited through snowballing, ensuring a wide age range for heterogeneity assessment. Heterogeneity was assessed according to biopsychosocial demographic characteristics related to income, education level, sex, and age. The 12th and 26th items of the draft instrument needed to be modified, and following the modifications; the pilot study was completed with a re-rating of the items using the same participants. The lowest item achieved a comprehension rate of 89.09%, whereas the overall scale achieved a comprehension rate of 93.7%, which was considered adequate. Upon completion of these steps, a detailed explanation of the steps was provided to the authors of the original study at the beginning of the construct validity phase.

### 2.4. Construct Validity Studies

Construct validity was assessed in two main phases: exploratory and confirmatory. The sample size was determined based on criteria specified in the literature, ensuring that the number of observations was at least 10 times the number of items [24]. Consequently, the observations were divided into two sections: the first 320 observations were allocated to the exploratory phase, which included item analysis and exploratory factor analysis (EFA), and the remaining 319 observations were designated for CFA. The exploratory phase, which included item analysis and EFA, aimed to identify structural properties within the dataset. In contrast, the confirmatory phase used CFA to validate the specific structure. The primary objective was to retest the measurement model using diverse responses, thereby confirming that the null hypothesis (H_0_) could not be rejected.

After the CFA, the validation phase was continued with assessments of convergent validity, discriminant validity, and known group validity using the entire dataset. Convergent validity examines whether similar variables capture the same construct, whereas discriminant validity measures the uniqueness of variables [29]. Convergent validity anticipates positive or negative correlations, whereas discriminant validity expects minimal or no significant correlations. Furthermore, discriminant validity was supported by the average variance extracted (AVE), which calculates the proportion of total variance explained by a factor across its items (observations). These forms of validity are crucial for determining the accuracy and reliability of a measurement tool [30,31]. Furthermore, known group validity, another confirmatory method, evaluates whether the measurement tool can accurately detect the theoretically expected differences between groups [32].

In the reliability analyses, the exploratory phase included item analysis and internal consistency evaluations using Cronbach’s alpha coefficients. In the confirmatory phase, composite reliability (CR), Cronbach’s alpha, Bland–Altman analysis for systematic error control, factor score agreement evaluations, and the assessment of %27 slice differentiation scores and their randomness were performed. The 27% slice differentiation method involves ranking individuals based on their total scores from the scale, then identifying the top 27% and bottom 27% groups. A *t*-test is performed to evaluate whether the items successfully differentiate between these two groups. This approach ensures that items align with the overall test performance, where higher scorers are expected to achieve higher item scores and lower scorers, lower item scores. Items that fail to show this pattern are considered to have weak discriminant power [33].

### 2.5. Research Hypotheses

The hypotheses were developed to assess variations in individual perceptions based on variables associated with access to health services. In this study, ’access to health services’ encompasses a broad range of healthcare services. However, it is important to note that this study was conducted in a tertiary healthcare institution, specifically a teaching and research hospital. This study examined several variables, namely, sex, education level, marital status, age group, kinship status with a healthcare professional, belief in the ease of access to health services, and presence of chronic diseases. The ability of these variables to reveal potential disparities in the results of the Perceived Access to Health Care Services Questionnaire is crucial for assessing the precision of the measurement tool. In this context, the following hypotheses were formulated:

1. Substantial differences in the results of the Perceived Access to Health Care Services Questionnaire are expected among variables such as sex, education level, marital status, age group, kinship status with a healthcare professional, belief in the ease of access to health services, and presence of chronic diseases.
2. A statistically significant and positive correlation is expected between the Perceived Access to Health Care Services Questionnaire scores and outpatient satisfaction, indicating the convergent validity of the questionnaire.
3. No statistically significant relationship is expected between the scores of the Culture of Healthy Living Scale and those of the Perceived Access to Health Care Service Questionnaire, indicating that the culture of healthy living is unrelated to access to health services, considering the possibility of a beta error.

### 2.6. Statistical Analysis

Data analysis was performed using Lisrel 8.51 and RStudio/2023.09.1+494. Intraclass correlation coefficient alpha and one-way analysis of variance (ANOVA) were used for model evaluation; sampling adequacy and factor analysis adequacy were assessed using the Kaiser–Meyer–Olkin (KMO) and Bartlett’s tests, respectively; moreover, principal component analysis was performed using the direct oblivion method. The independent samples *t*-test, Welch’s two-sample *t*-test, and ANOVA were used for comparative analyses. The Tukey HSD and Games–Howell tests were used for post hoc analysis. Bland–Altman analysis was performed to control for systematic errors. Alpha and CR coefficients were used in reliability measurements, and the average variance extraction coefficient was used in validity measurements. Pearson’s correlation coefficient was used for correlational analyses, with assessments made at the 95% confidence level.

### 2.7. Research Limitations and Inclusion and Exclusion Criteria

The potential limitations of this study include the prospect that increasing the sample size and scope may increase the generalizability of the findings. In particular, the applicability of the results derived from a study focused on outpatients from a specific hospital may be limited when extrapolating to the broader context of perceptions regarding access to health services throughout Turkey. Furthermore, this study focused exclusively on the adult population, excluding individuals under 18 years of age. Thus, there may be differences in perceptions of access to health services among young individuals, and the results of this study cannot be broadly extrapolated to these age groups. Another plausible limitation of this study is related to the sampling method. Despite the use of simple random sampling, this method does not always ensure that the sample group is fully representative of the general population.

The inclusion criteria were as follows: adults who received outpatient treatment at Kanuni Sultan Süleyman Training and Research Hospital in Istanbul within the specified time frame and who could understand and speak Turkish, the survey language. In contrast, the exclusion criteria were as follows: individuals who were hospitalized at the time of the survey, those who lacked the physical or mental capacity to complete the survey, and other specific circumstances that did not meet this study’s criteria.

### 2.8. Ethical Approval and Consent to Participate

This study was reviewed and approved by the Ethics Committee of Istanbul Arel University (Ethical Code: E-52857131-050.06.04-258830, Date: 10 March 2023, Decision No: 2023/05) following the ethical principles of the Declaration of Helsinki: Ethical Principles for Medical Research Involving Human Subjects. Written informed consent was obtained from each participant. We certify that all experiments were conducted in accordance with relevant guidelines and regulations.

## 3. Results

According to this study’s results, the sex distribution was balanced with 50% females and 50% males, and the predominant education level was a bachelor’s degree, representing 38.8% of the participants. A significant proportion of the participants (63.7%) were married, and most participants fell within the age range of 40–65 years, representing 67.8% of the total. The ’single’ category also includes individuals who, in addition to being single, are divorced or widowed. Approximately one-third of the participants reported the presence of an active healthcare professional in their close circle. Regarding access to health services, slightly less than half of the participants found access easy, whereas 37.1% found it difficult. Furthermore, 73.6% of the participants reported no chronic diseases.

The first 320 participants were assigned to the exploratory phase of the scale, and the remaining 319 were assigned to the confirmatory phase.

The scale underwent reliability analysis, and the alpha coefficient of the 30-item scale was 0.936 in the first phase. Further investigation revealed that removing item 17 increased the alpha value to 0.937, with a similar increase observed for removing items 18 and 19, resulting in alpha values of 0.937 and 0.943, respectively. Therefore, these three items were removed. Table 1 presents the results of the reliability analysis after removing these items.

Table 1 presents the initial results of the reliability analysis and intraclass correlation. The Cronbach’s alpha coefficient of 0.946 is considered remarkably high. The Single Measures value, which indicates the reliability for each individual, was 0.361, whereas the Average Measures value, which indicates the reliability of the average scores within the group, was 0.938. In the one-way random model, the F statistic value was 16.240, with a *p*-significance value for the model of <0.001. Within the 27-item model, the mean score was 89.83 ± 18.21.

**Figure 1:**
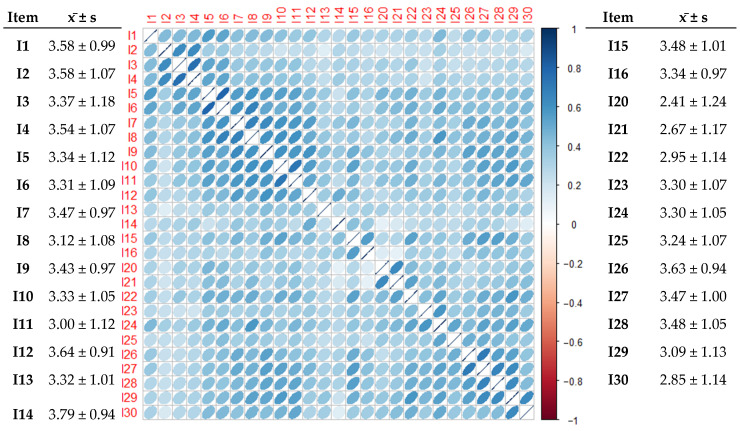
Item analysis of the scale.

According to the item analysis table, three items, particularly items 14, 16, and 20, exhibited inconsistency with the overall measurement. In particular, item 14 had nine scores with a correlation of <0.3, whereas items 16 and 20 each had five scores with a correlation of <0.3. Consequently, these items were removed to improve the overall measurement consistency. Before removal, item 14 had the highest mean score (3.79 ± 0.94), and item 20 had the lowest mean score (2.41 ± 1.24). After excluding these three items, item 12 had the highest mean score (3.64 ± 0.91), whereas item 30 had the lowest mean score (2.85 ± 1.14), as shown in the model (Figure 1).

When evaluating Cronbach’s alpha values with the stepwise removal of the items identified for potential exclusion, it was observed that the alpha value decreased when item 20 was removed but remained stable when items 14 and 16 were removed. Considering that items 14 and 16 were selected for removal because of low correlation, item 20 was not removed from the scale to maintain internal consistency. Consequently, items 14 and 16 were removed, resulting in a Cronbach’s alpha of 0.946 for the remaining 25 items. At this point, the mean score was 82.68 ± 17.30. EFA was then performed.

In Table 2, following the principal component analysis used in the EFA, the rotation was performed using the direct oblivion method, which yielded results after 13 iterations.

Following the initial EFA, items 6 and 22 were removed. The reason for removing item 6 was its loading on the first and second factors with a difference of only 0.078, a value of <0.10, indicating its appropriateness for removal. Likewise, item 22 was removed because of its loading on the third and fourth factors, with a difference of only 0.073. In the final EFA, the KMO criterion for the factor analysis exhibited perfect compatibility at 0.936. Bartlett’s test of sphericity indicated a significant relationship between the variables, confirming the feasibility of factor analysis. The final factor analysis included 23 items that explained 62.904% of the cumulative variance. The variance explained according to the factors, with the items distributed based on the factor loads, was as follows (Table 2):

- The first factor was determined by items I7, I8, I9, I10, I11, I12, and I13.
- The second factor was determined by items I2, I3, and I4, explaining 8.106% of these data.
- The third factor was determined by items I20 and I21, explaining 5.183% of these data.
- The fourth factor was determined by items I23, I24, I25, I26, I27, I28, I29, and I30, explaining 5.169% of these data.

After the EFA, the alpha coefficient was recalculated and found to be 0.939 for the remaining 23 items, which marked the end of the exploratory phase. In the confirmatory phase, a separate sample group of 319 individuals, separate from the original 320 individuals involved in the EFA, was analyzed.

In the confirmatory phase, structural equation modeling was used for factor analysis. The results of the structural equation modeling showed that the model has a generally good fit for these data. The χ^2^/df ratio of 2.202 is within the acceptable limits. The RMSEA value of 0.061 indicates that the model provides an acceptable fit, and the GFI value of 0.90 and the AGFI value of 0.86 are also considered acceptable. Furthermore, an NNFI (TLI) index of 0.94 and an NFI index of 0.91 indicate that the model has an acceptable fit. In particular, a CFI index of 0.95 indicates an excellent fit of the model (Table 3).

**Figure 2:**
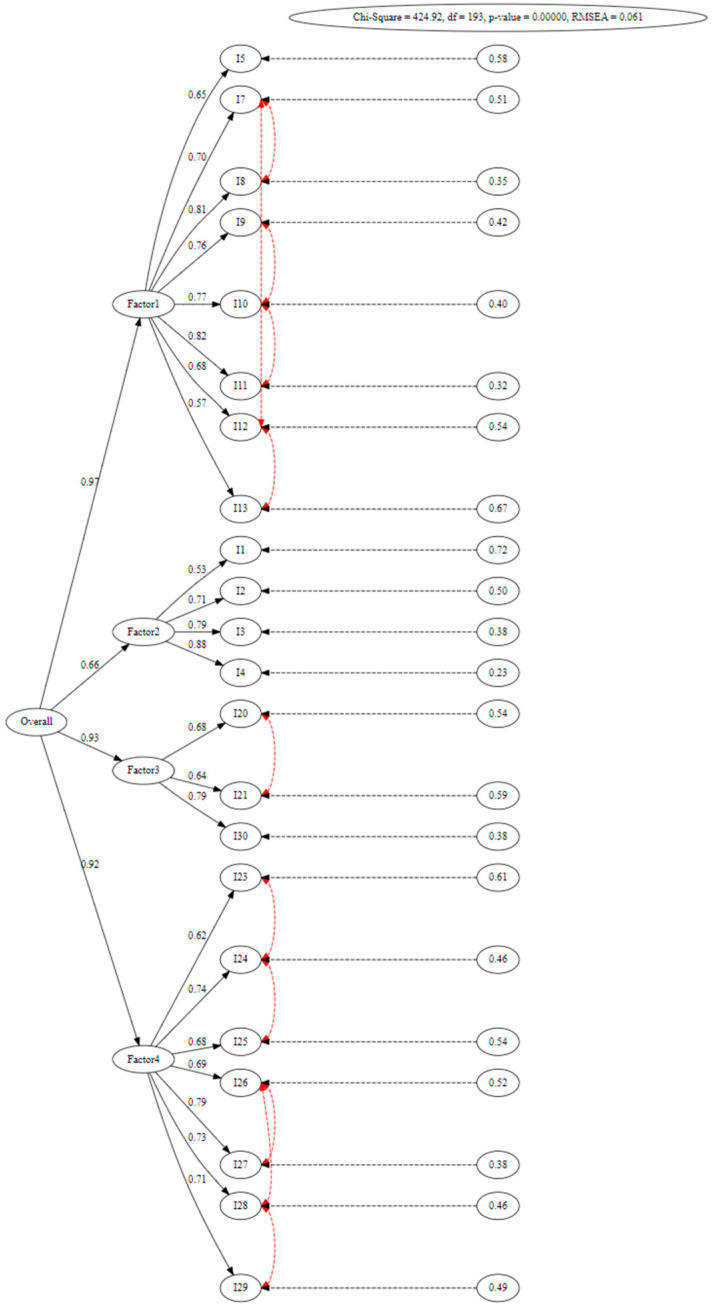
Path diagram of the model.

The path diagram of the model illustrates the correlation of the error terms among the specific measurements within the four factors. Red lines represent error associations. The model includes error covariances between I28 and I29, I26 and I27, I23 and I24, I12 and I13, I10 and I11, I7 and I8, I9 and I10, I7 and I12, I27 and I28, I20 and I21, I24 and I25, and I26 and I28. These error covariances were based on the assumption that measurement errors are correlated with certain observed variables and are, therefore, selectively applied among specific observed variables within the factors. The path diagram for the second-order confirmatory factor analysis demonstrated a good fit and yielded successful results according to the index ranges in Table 3 (Figure 2).

The CR scores for all factors and the Access score were above the acceptable threshold of 0.7, indicating high internal consistency, with Factor 2 (0.902) and Access (0.942) demonstrating the strongest reliability. Although the AVE values for all factors and Access were slightly below the recommended threshold of 0.5, they ranged between 0.529 and 0.561, suggesting moderate construct validity. The Welch two-sample *t*-test results for splitting these data into top and bottom 27% slices exhibited significant discrimination across all factors and Access, with extremely high t-values (e.g., −43.273 for Access) and *p*-values below 2.2 × 10^−16^, confirming strong discriminatory capacity (Table 4).

**Figure 3:**
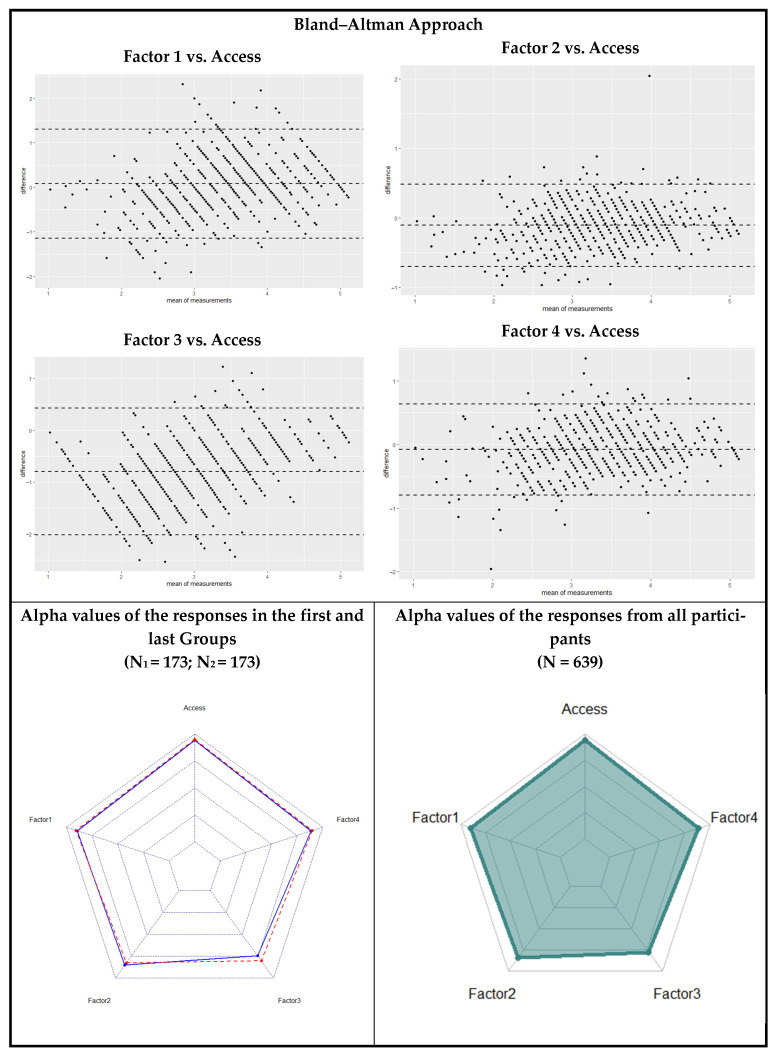
Systematic Errors in Factor Consistency Analyses and Radar Charts Displaying Alpha Coefficients.

Table 4 presents the separation of 173 participants in the 27% lowest-scoring percentile and 173 participants in the 27% highest-scoring percentile based on factors and the overall score. To investigate whether this separation was random, Cronbach’s alpha reliability coefficients of two randomly selected groups of 173 participants were compared using the radar graphs in Figure 3. The alpha coefficients of the final scale, which were calculated for all participants, are also presented in radar graph format. Furthermore, the Bland–Altman approach was used to test the alignment of the factors with the Access overall score and to determine whether systematic bias was present. Table 5 examines the similarity of responses from two randomly selected groups of 173 participants using Student’s *t*-test and correlation analysis. Randomness was ensured by forming two groups: the first 173 participants and the last 173 participants, based on the order of survey completion.

Figure 3 presents a visual representation of systematic errors identified in factor consistency analyses. Additionally, radar charts illustrate the alpha coefficients for each factor, providing a clear overview of internal consistency across dimensions. These visual tools enhance the interpretability of the results and highlight areas where factor reliability may require further investigation.

The Bland–Altman approach was used to examine the similarity between the averages of the scores derived from the factors and the overall score. All factors included 0 within the 95% confidence interval, and the middle line was very close to 0, indicating that, on average, the factors demonstrated consistent alignment with the overall score. In contrast, Cronbach’s alpha coefficients derived from the responses of the first 27% of participants (173 individuals) are shown in blue in the radar graph in the bottom-left corner. These values were 0.894 for Factor 1, 0.851 for Factor 2, 0.745 for Factor 3, 0.885 for Factor 4, and 0.942 for Access. For the last 27% (173 participants), these values are represented by a dashed red line and were 0.906 for Factor 1, 0.828 for Factor 2, 0.802 for Factor 3, 0.899 for Factor 4, and 0.950 for Access. These results were considered highly similar, indicating sufficient reliability for Factor 3 and high reliability for the remaining factors. The final Cronbach’s alpha coefficients of the complete measurement tool, which were calculated for all participants, are displayed in the bottom-right corner of Figure 3. These values were 0.899 for Factor 1, 0.825 for Factor 2, 0.773 for Factor 3, 0.892 for Factor 4, and 0.943 for Access, representing the entire scale (Figure 3).

In Table 5, the responses of two randomly selected groups, each comprising 173 participants, were compared across the five factors using independent samples *t*-tests. The results indicated no significant differences between the first and last groups for any of the factors (*p* > 0.05). Accordingly, the discriminant validity established in Table 4 was confirmed not to be random because similarity was observed in the randomly selected groups, whereas the separation of the lowest and highest scoring groups was demonstrated to not occur by chance.

Table 6 shows the known group validity and the convergent validity of the measurement tool assessed with 639 participants. Of the 639 participants, 320 (50.1%) were female and 319 (49.9%) were male. Regarding marital status, 407 (63.7%) were married, and 232 (36.3%) were single. Regarding age, 187 (29.3%) aged ≤ 39 years, 433 (67.8%) aged between 40 and 65 years, and 19 (3.0%) aged ≥ 66 years. The mean age was 44.95 ± 11.20 years (range, 18–76 years). Of the participants, 434 (67.9%) had no relatives who were healthcare professionals, whereas 205 (32.1%) had relatives who were active healthcare professionals. Regarding educational status, 25 (3.9%) had primary and secondary education, 93 (14.6%) had high school education, 87 (13.6%) had a college education, 248 (38.8%) had a bachelor’s degree, and 186 (29.1%) had a master’s degree/doctorate. Regarding access to health services, 300 individuals (46.9%) reported that they could easily access the health services they wanted, 102 individuals (16.0%) were undecided, and 237 individuals (37.1%) stated that they could not access them. Regarding health status, 470 (73.6%) participants reported having no chronic diseases, whereas 169 (26.4%) reported having a chronic disease.

In the known group validity section, significant differences were found in the variables of age and belief in easy access to health services. Differences were observed in Factor 1, Factor 3, Factor 4, and the total Access score based on age. Individuals aged ≤39 years had higher scores on Factor 1, Factor 3, and Access than those in the higher age groups. For Factor 4, individuals aged ≤39 years scored higher than those aged 40–65 years. Those who believed that it was easy to access health services scored higher on all factors than those who were unsure or responded negatively. Moreover, those who were unsure scored higher on all factors than those who answered negatively.

Factor 1 exhibited positive and moderate to high correlations with all components of the Outpatient Satisfaction Scale (e.g., r = 0.770 with Appointment), whereas Factor 2 generally showed weak to moderate correlations (e.g., r = 0.356 with Effective Examination). Factor 3 demonstrated moderate to highly positive correlations (e.g., r = 0.693 with Staff Attitude), and Factor 4 exhibited high correlations with all components (e.g., r = 0.660 with Waiting Time). In contrast, Access had high positive correlations with all components (e.g., r = 0.712 with Overall Satisfaction). For the “Crisis with Individual Action” and “Judgment” subscales of the Healthy Living Culture Scale, divergent validity analysis revealed generally low and insignificant correlations with the four-factor scores and the total score of the Perceived Access to Health Care Questionnaire. The results suggest that because the relationships between these variables were insignificant, the variables were independent of each other, indicating divergent validity (Table 7).

## 4. Discussion

The Turkish validity and reliability study of the Perceived Access to Health Care Questionnaire identified four main factors associated with access to and quality of health services. After evaluating the questions under the structure formed by the items and categorized into four latent variables, naming the first factor as “Acceptability and Affordability” was considered appropriate. This factor includes crucial elements such as the knowledge and skills of healthcare professionals, their interaction with patients, the quality of treatment, and the cost of services. It is considered to reflect both the acceptability and cost-effectiveness of health services. A study conducted in South Africa highlighted the importance of acceptability and affordability, particularly income, in the perception of health services and access to needed care [34].

After examining the items of the second factor, it was named “Availability”. This factor focuses on the ease of access to health services, availability of services, and geographic proximity. A study published in 2020 highlighted the importance of geographic access to health services in the United States over 15 years, revealing its association with improved outcomes in chronic disease management and revealing that regions with lower socioeconomic status had lower utilization of healthcare services [35].

The third factor was related to “Accommodation”. This refers to how health services are organized based on patients’ needs and socioeconomic status and includes appointment processes and waiting times. One study recommended adjusting for socioeconomic status to ensure patient-centered healthcare and equal quality of healthcare and found that socioeconomic factors have a significant impact on patient’s perceptions of the quality of healthcare, particularly in terms of waiting time, the environment of the outpatient service, and the quality of healthcare provided by healthcare providers [36].

Finally, Factor 4 was named “Awareness” because it covers the patient’s level of knowledge about healthcare services and the comprehensibility of training and information provided by healthcare professionals. In a study on individuals with breast cancer, patient activation was found to be associated with cancer literacy and awareness [37]. Another study found that increasing health literacy positively affects chronic disease management, awareness of healthy lifestyles, reduction in healthcare expenditures, premature death, and disability, among others, and that it can reduce the demand for healthcare services [38].

Following remodeling performed with the permission of the authors of the original scale, it was determined that these four factors represent different dimensions of access to health services and together contribute to an overall score. Furthermore, the total score was named “Perceived Accessibility”, recognizing that these factors together provide a comprehensive perspective on the quality and accessibility of health services. In another methodological scale development and cross-sectional application study on access to healthcare, consumers’ perceived access was found to have a positive correlation with health sensitivity and overall health outcomes [39]. An investigation of perceived access to health services during the COVID-19 pandemic in Germany indicated that perceived access decreased under restrictions, and measures such as increased use of telemedicine and appointment systems were identified as strategies to maintain access to health services [40].

A study by Tadiri et al. [41] highlighted social factors such as gender equality and marital status as potential determinants of access to health services while excluding education as a significant factor. In parallel with these findings, this study did not find a significant relationship between education and access to health services. However, in contrast to the findings of Tadiri et al. [41], sex and marital status had no direct impact on access to health services. This difference may be due to the cultural differences in healthcare systems and social life between Turkey and Canada/Australia. For instance, gender equality is more established in Canada and Australia compared with Turkey, where traditional gender roles may influence perceptions of access to healthcare. Additionally, marital status may carry different societal implications in Turkey, potentially affecting healthcare accessibility less prominently. Furthermore, education level has been suggested to play a similar role in these three countries. However, a longitudinal study in China, conducted with 14 years of data, found age-related differences in access to health services, with approximately 6.5% of the elderly reporting lower perceived access to health services [42]. These findings suggest an age-related escalation in perceived inequalities in access to health, which is consistent with the findings of this study. Likewise, the perception of access to health services decreased with increasing age in our study.

Considering that having a relative who is a healthcare professional or having a chronic disease may significantly influence perceptions of access to health services, it was included in the assessment of the validity of known groups. This is because, considering the ratio of physicians to the population in Turkey (2.05 per 1000 individuals in 2020) and the recent decline in the number of physicians in Turkey, securing a timely appointment may be a challenge [43,44]. Thus, it was hypothesized that this circumstance may act as a differentiating factor in Turkey, where interpersonal connections are commonly used for quick access to health services [45]. Furthermore, the frequency of the need felt by individuals with chronic diseases to access health services could be a differentiating factor in this context. However, our results differed from those expected. This could be attributed to the involvement of broader and more complex factors that influence access to health services in Turkey. A study on the factors that influence elderly individuals’ access to health services during the COVID-19 pandemic highlighted the multifaceted nature of access to health services, where psychological, physical, and economic barriers, along with broader societal factors such as cultural issues and legal barriers, collectively contribute to the perceptions and realities of access to health services [46]. At this point, it was suggested that perceived access to health services is correlated with aspects such as satisfaction with health services, and positive relationships were found between all the factors and the Outpatient Satisfaction Scale and its subscales. Another study in Wisconsin found a correlation between limited access to health services for patients with hearing loss and satisfaction with healthcare services [20]. A study conducted by the Veterans Health Administration in the United States found significant relationships between quality, perceived access, and satisfaction when assessing the mental health of outpatient veterans [47].

In some cases, the scale revealed differences and relationships between groups, whereas, in other cases, it showed results contrary to expectations. Studies have highlighted the complexity of the factors that influence perceptions of access to health services [48,49]. However, the development and widespread use of measurement tools can facilitate a deeper understanding of factors, such as satisfaction and the extent of perceived access.

## 5. Conclusions

Access to health services is a determinant that significantly affects the overall health status of individuals and communities. Assessing access to health services is central to promoting an equitable and just health system. Thus, questionnaires that measure perceived access to healthcare, along with alternative hypotheses that support the validity of these questionnaires, play a critical role in developing health policies and practices. The linguistic and cultural validity studies, which include translations, pilot testing, and the validation of the structural model, represent a critical step in ensuring the validity and reliability of the questionnaire in the local context. It is posited that the questionnaire effectively captures respondents’ perceptions of access to health services and provides a valuable foundation for researchers working in this area.

This study relies solely on survey data and does not include objective data on healthcare utilization beyond participants’ perceptions of access to health services. This study is limited to individuals aged 18–65, excluding those over 65 years old, which restricts the generalizability of the findings to broader age groups. Additionally, this study employed a snowball sampling method, which contributed to achieving a heterogeneous sample but carries the risk of overrepresentation of certain groups due to its reliance on social networks. Future research should address these limitations by incorporating a broader age range and employing more representative sampling methods. For the survey, see Appendix A.

## Figures and Tables

**Table 1:** First reliability and correlation analysis results.

*n*	Alpha	Single Measures	Average Measures	Mean ± SD	*F*	*p*
27	0.946	0.361	0.938	89.83 ± 18.21	16.240	<0.001

**Table 2:** Exploratory factor analysis.

Initial Analysis						Final Analysis				
Item	1	2	3	4		Item	1	2	3	4
**I1**	*0.277*	***0.493***	*0.040*	*0.044*		**I1**	*0.263*	***0.497***	*0.045*	*0.057*
**I2**	*−0.201*	***0.899***	*−0.104*	*0.158*		**I2**	*−0.190*	***0.900***	*−0.091*	*0.134*
**I3**	*0.098*	***0.822***	*0.072*	*−0.078*		**I3**	*0.114*	***0.837***	*0.074*	*−0.103*
**I4**	*0.105*	***0.831***	*0.003*	*−0.021*		**I4**	*0.107*	***0.838***	*0.021*	*−0.038*
**I5**	***0.502***	*0.377*	*0.277*	*−0.107*		**I5**	***0.467***	*0.374*	*0.248*	*−0.046*
**I6**	***0.454***	*0.376*	*0.282*	*0.054*		**I7**	***0.735***	*0.107*	*−0.055*	*0.075*
**I7**	***0.747***	*0.098*	*−0.039*	*0.068*		**I8**	***0.607***	*0.116*	*0.202*	*0.076*
**I8**	***0.627***	*0.110*	*0.218*	*0.041*		**I9**	***0.690***	*0.096*	*−0.094*	*0.190*
**I9**	***0.694***	*0.092*	*−0.126*	*0.213*		**I10**	***0.828***	*0.002*	*0.076*	*0.004*
**I10**	***0.798***	*−0.025*	*0.064*	*0.054*		**I11**	***0.724***	*0.015*	*0.185*	*0.029*
**I11**	***0.703***	*−0.006*	*0.170*	*0.059*		**I12**	***0.685***	*0.074*	*−0.078*	*0.073*
**I12**	***0.679***	*0.061*	*−0.095*	*0.104*		**I13**	***0.425***	*−0.034*	*0.246*	*0.056*
**I13**	***0.410***	*−0.042*	*0.188*	*0.091*		**I15**	*0.350*	*0.083*	*−0.151*	***0.470***
**I15**	*0.302*	*0.071*	*−0.119*	***0.523***		**I20**	*0.087*	*0.096*	***0.770***	*0.018*
**I20**	*0.063*	*0.075*	***0.763***	*0.002*		**I21**	*0.064*	*0.030*	***0.741***	*0.124*
**I21**	*0.033*	*0.009*	***0.773***	*0.101*		**I23**	*−0.122*	*0.025*	*0.202*	***0.678***
**I22**	*0.177*	*0.043*	***0.408***	*0.335*		**I24**	*−0.030*	*0.166*	*0.302*	***0.583***
**I23**	*−0.134*	*0.037*	*0.222*	***0.655***		**I25**	*−0.159*	*0.111*	*0.155*	***0.683***
**I24**	*−0.031*	*0.173*	*0.326*	***0.543***		**I26**	*0.192*	*0.022*	*−0.211*	***0.744***
**I25**	*−0.137*	*0.135*	*0.168*	***0.624***		**I27**	*0.221*	*−0.018*	*−0.108*	***0.762***
**I26**	*0.196*	*0.042*	*−0.198*	***0.733***		**I28**	*0.218*	*−0.030*	*−0.018*	***0.682***
**I27**	*0.203*	*−0.008*	*−0.093*	***0.771***		**I29**	*0.267*	*−0.056*	*0.192*	***0.547***
**I28**	*0.188*	*−0.025*	*−0.008*	***0.704***		**I30**	*0.275*	*−0.061*	***0.407***	*0.279*
**I29**	*0.229*	*−0.063*	*0.213*	***0.566***	**KMO: 0.936; Bartlett’s χ^2^: 4254.75; *p* < 0.001**					
**I30**	*0.256*	*−0.070*	***0.420***	*0.268*	**Component 1: 10.078; 43.817% Component 2: 1.864; 8.106%**		**Component 3: 1.337; 5.183% Component 4: 1.189; 5.169%**			

Bold is the highest factor load for item. In the following items, different factors are represented by different colors. Additionally, the items in red are the ones that have been removed.

**Table 3:** Confirmatory factor analysis goodness of the fit indices.

Indices	Perfect Fit	Acceptable Fit	Conclusion	
			Value	Fitness
χ^2^/df	0 ≤ χ^2^/df ≤ 2	2 ≤ χ^2^/df ≤ 3	2.202	Acceptable
RMSEA	0 < RMSEA ≤ 0.05	0.05 < RMSEA ≤ 0.08	0.061	Acceptable
GFI	0.95 ≤ GFI ≤ 1.00	0.90 ≤ GFI < 0.95	0.90	Acceptable
AGFI	0.90 ≤ AGFI ≤ 1.00	0.85 ≤ AGFI < 0.90	0.86	Acceptable
NNFI (TLI)	0.95 ≤ NNFI (TLI) ≤ 1.00	0.90 ≤ NNFI (TLI) < 0.95	0.94	Acceptable
NFI	0.95 ≤ NFI ≤ 1.00	0.90 ≤ NFI < 0.95	0.91	Acceptable
CFI	0.95 ≤ CFI ≤ 1.00	0.90 ≤ CFI < 0.95	0.95	Perfect

χ^2^/df: chi-square/degrees of freedom; RMSEA: root mean square error of approximation; GFI: goodness of fit index; AGFI: adjusted goodness of fit index; NNFI: non-normed fit index; TLI: Tucker–Lewis Index; NFI: normed fit index; CFI: comparative fit index.

**Table 4:** Final validity, reliability, and group comparison scores based on 27% slices.

	***n***	**Factor 1**	**Factor 2**	**Factor 3**	**Factor 4**	**Access**
**Composite reliability**	639	0.831	0.902	0.783	0.887	0.942
**Average variance ext.**	639	0.561	0.539	0.552	0.533	0.529
**Welch two-sample *t*-test comparison for splitting into 27% slices**						
	***n***	**Factor 1**	**Factor 2**	**Factor 3**	**Factor 4**	**Access**
***t*** **-value**	346	−20.984	−32.656	−24.617	−30.454	−43.273
***p*** **-value**		2.2 × 10^−16^	2.2 × 10^−16^	2.2 × 10^−16^	2.2 × 10^−16^	2.2 × 10^−16^

**Table 5:** Student *t*-rest results for similarity across randomly selected groups.

	*n*	$\bar{x}$ ± s	*t*	*p*
**Factor 1**				
First group	173	26.67 ± 5.88	−0.367	0.714
Last group	173	26.91 ± 6.4		
**Factor 2**				
First group	173	14.11 ± 3.47	−0.278	0.781
Last group	173	14.21 ± 3.48		
**Factor 3**				
First group	173	7.78 ± 2.8	−0.856	0.392
Last group	173	8.05 ± 3.1		
**Factor 4**				
First group	173	26.62 ± 6.03	−0.775	0.439
Last group	173	27.14 ± 6.46		
**Access**				
First group	173	75.18 ± 15.55	−0.649	0.517
Last group	173	76.32 ± 17.05		

*t*: Independent samples *t*-test value.

**Table 6:** Known group validity, convergent validity, and divergent validity of the measurement tool.

		Factor 1	Factor 2	Factor 3	Factor 4	Access
	***n***	**$\bar{x}$ ± s**	**$\bar{x}$ ± s**	**$\bar{x}$ ± s**	**$\bar{x}$ ± s**	**$\bar{x}$ ± s**
**Sex**						
Female	320	26.65 ± 6.01	13.98 ± 3.52	7.77 ± 2.87	27.08 ± 6.06	75.47 ± 15.91
Male	319	26.63 ± 6.63	14.17 ± 3.5	8.1 ± 3.03	26.92 ± 6.53	75.82 ± 16.71
*t*		0.046	−0.700	−1.422	0.314	−0.268
*p*		0.963	0.484	0.156	0.754	0.789
**Education level**						
Elementary/middle school	25	27.4 ± 7.96	13 ± 5.2	9.08 ± 3.04	27.84 ± 7.12	77.32 ± 21.25
High school	93	25.68 ± 7.22	13.41 ± 3.6	7.85 ± 2.97	26.08 ± 7.1	73.01 ± 18.53
Associate degree	87	27.68 ± 6.45	13.84 ± 3.37	7.91 ± 3.08	27.6 ± 6.37	77.02 ± 16.68
Bachelor’s degree	248	26.68 ± 5.95	14.23 ± 3.36	7.63 ± 2.89	27.15 ± 6.04	75.69 ± 14.99
Graduate	186	26.47 ± 6	14.44 ± 3.4	8.24 ± 2.91	26.89 ± 6.07	76.04 ± 15.91
*F*		1.253	2.173	2.148	0.856	0.855
*p*		0.287	0.071	0.073	0.490	0.491
*Post hoc*		-	-	-	-	-
**Marital status**						
Married	407	26.44 ± 6.33	14.22 ± 3.45	7.9 ± 2.97	26.91 ± 6.32	75.47 ± 16.25
Single	232	26.99 ± 6.3	13.81 ± 3.6	7.98 ± 2.93	27.16 ± 6.26	75.95 ± 16.42
*t*		−1.066	1.401	−0.306	−0.487	−0.355
*p*		0.287	0.162	0.760	0.627	0.723
**Age**						
≤39 years _a_	187	27.84 ± 6.32	14.23 ± 3.57	8.5 ± 2.91	28.01 ± 6.61	78.58 ± 16.97
40–65 years _b_	433	26.22 ± 6.24	14.06 ± 3.43	7.73 ± 2.94	26.62 ± 6.13	74.63 ± 15.82
≥66 years _c_	19	24.32 ± 6.58	12.84 ± 4.57	6.84 ± 2.83	25.84 ± 6.1	69.84 ± 17.07
*F*		**5.723**	1.362	**5.786**	**3.517**	**5.129**
*p*		**0.003**	0.257	**0.003**	**0.030**	**0.006**
*Post hoc*		**a > b, c**	-	**a > b, c**	**a > b**	**a > b, c**
**Presence of a relative who is an active health worker**						
No	434	26.55 ± 6.52	14.08 ± 3.53	7.99 ± 2.98	26.78 ± 6.49	75.4 ± 16.8
Yes	205	26.82 ± 5.9	14.05 ± 3.48	7.8 ± 2.88	27.48 ± 5.86	76.16 ± 15.21
*t*		−0.511	0.115	0.743	−1.311	−0.564
*p*		0.610	0.909	0.458	0.190	0.573
**Beliefs about easy access to health services**						
Yes	300	29.92 ± 5.43	15.69 ± 2.77	9.58 ± 2.65	29.54 ± 5.72	84.73 ± 14.09
Not sure	102	25.75 ± 4.36	13.75 ± 2.74	7.69 ± 2.1	26.61 ± 4.38	73.8 ± 9.6
No	237	22.87 ± 5.86	12.16 ± 3.65	5.95 ± 2.3	23.96 ± 6.32	64.94 ± 14.35
*F*		**112.699**	**84.897**	**147.533**	**62.238**	**141.612**
*p*		**<0.001**	**<0.001**	**<0.001**	**<0.001**	**<0.001**
*Post hoc*		**a > b, c** **b > c**	**a > b, c** **b > c**	**a > b, c** **b > c**	**a > b, c** **b > c**	**a > b, c** **b > c**
**Presence of a chronic disease**						
No	470	26.71 ± 6.26	14.05 ± 3.49	8.02 ± 2.93	27.16 ± 6.18	75.94 ± 16.13
Yes	169	26.43 ± 6.52	14.13 ± 3.55	7.69 ± 3	26.58 ± 6.61	74.82 ± 16.8
*t*		0.509	−0.251	1.258	1.019	0.764
*p*		0.611	0.802	0.209	0.309	0.445

*n* = 639; *t*: independent samples *t*-test; *F*: one-way analysis of variance. ^a^: ≤39 years; ^b^: 40–65 years; ^c^: ≥66 years.

**Table 7:** Correlation coefficients between Perceived Access to Health Care scores, Outpatient Satisfaction Scale, and Healthy Living Culture Scale.

**Convergent Validity**														
	**Outpatient Satisfaction Scale**													
**Perceived Access to Health Care**	**Appointment**		**Effective Examination**			**Staff Attitude**			**Waiting Time**				**Overall Satisfaction**	
	**r**	***p***	**r**		***p***	**r**	***p***		**r**	***p***			**r**	***p***
**Factor 1**	0.586	<0.001	0.770		<0.001	0.631	<0.001		0.602	<0.001			0.662	<0.001
**Factor 2**	0.449	<0.001	0.494		<0.001	0.356	<0.001		0.379	<0.001			0.482	<0.001
**Factor 3**	0.701	<0.001	0.682		<0.001	0.547	<0.001		0.693	<0.001			0.622	<0.001
**Factor 4**	0.637	<0.001	0.804		<0.001	0.708	<0.001		0.631	<0.001			0.660	<0.001
**Access**	0.697	<0.001	0.839		<0.001	0.694	<0.001		0.684	<0.001			0.712	<0.001
**Divergent Validity**														
	**Healthy Living Culture Scale**													
**Perceived Access to Health Care**	**Crisis with individual action**						**Judgment**							
	**r**			***p***			**r**					***p***		
**Factor 1**	−0.002			0.959			−0.026					0.519		
**Factor 2**	0.005			0.891			−0.032					0.412		
**Factor 3**	0.003			0.949			0.005					0.904		
**Factor 4**	−0.029			0.472			−0.041					0.303		
**Access**	−0.010			0.789			−0.036					0.366		

*n* = 639; r: Pearson’s correlation.

## Data Availability

These original data presented in this study are openly available at https://www.dosya.tc/server/1wsmjk/verilertumsonanaliz.zip.html, (accessed on 8 February 2025).

## Appendix A

**Table A1:** Items of the scale.

Adaptation	Original Version
1. İhtiyaç duyduğum hizmetler sağlık kuruluşlarında sunulmaktadır.	The services I need are provided at the health center.
2. Sağlık kuruluşlarının evime olan uzaklığı uygundur.	The distance from the health centers to my house is appropriate.
3. Sağlık kuruluşlarına uygur sürede ulaşabiliyorum.	The time required to reach the health center is appropriate.
4. Sağlık kuruluşlarına kolaylıkla gdp gelebiliyorum.	Getting to and from the health center is easy for me.
5. İhtiyaç duyduğum sağlık hizmetlerini sağlık kuruluşlarından alabilmekteyim.	The health services (immunization, medical visit, family planning, mother and childcare, injections, etc.) I need are provided at the public health center.
6. Sağlık kuruluşlarının imkanlarını sağlık ihtiyaçlarımı karşılamaktadır.*	The facilities of the health center meet the health needs of the clients.
7. Sağlık personeli, ihtiyaçlarımı karşılamak için gerekli bilgi ve beceriye sahiptir.	Health staff is tailored to the number of clients and their needs.
8. Sağlık kuruluşlarında sunulan hizmetlerin kalitesi yeterlidir.	The quality of services provided in the health center is acceptable.
9. Sağlık personeli, benim ihtiyacımla ilgili kaynakları benim için kullanır.	The health center staff meets the needs of the clients in various ways, such as being introduced to the community resources.
10. Sağlık personal beni dictate dinger.	Health workers listen carefully to what I have to say.
11. Sağlık çalışanları bana yeterli zamanı ayırır.	The health workers give me enough time.
12. Sağlığım ihtiyacımla ilgili tedavi ekibinin (doktor, hemşire, ebe, vb) açıklamalarına güvenirim.	I trust the statements of the treatment team (doctor, nurse, midwife, etc.) about my health and illness
13. Benimle aynı cinsiyette olan sağlık personelinden hizmet alma isteğim dikkate alınır.	My request for same sex health care professionals is taken into account.
14. Sağlık merkezinde yapılan rahim veya kolon kanseri gibi test ve tarama hizmetlerini kabul ederim.*	I accept screening services such as cervical and colon cancer at this center.
15. Sağlık çalışanları genellikle saygılıdır.	The treatment team at the health center is respectful.
16. Sağlık çalışanlarıyla benim kültürel özelliklerimiz benzerdir.*	Health workers (doctors, nurses, midwives, etc.) are familiar with the culture of clients and communicate with them appropriately.
17. Her türlü sağlık ihtiyacım için öncelikle Aile Hekimime giderim.*	To solve my health problem, I first see a general practitioner.
18. Uzman hekimlere Aile Hekimimin önerisiyle giderim.*	With the guidance of a general practitioner, I use specialized and sub-specialized services.
19. Sağlık ihtiyaçlarımı gidermek için karşılaştığım parasal sorunlar, hizmeti elde etmemde engel oluşturmaktadır.*	Cost is a serious barrier to using health care.
20. Sağlık kuruluşlarından kolaylıkla randers alabilmekteyim.	It is easy to make an appointment at a health center.
21. İhtiyaç duyduğum sağlık hizmetini almak için bekleme süresi Kabul edilebilirdir.	The expected time to receive the services I need is appropriate.
22. Sağlık ihtiyacım ile ilgili gelişmeler hakkında tedavi ekibiyle iletişim kurabilmekteyim.*	I can discuss health issues and changes in my condition over the phone (in person) with the treatment team (doctor, nurse, midwife, etc.).
23. Kamu sağlık kuruluşlarındaki çalışma saatleri, hizmet alabilmem için uygundur.	The working hours of the public health center are suitable for receiving services from these centers.
24. Sağlık kuruluşlarının fiziksel özellikleri ihtiyaç duyduğum hizmetler için uygundur.	The physical space of the health center is suitable for receiving services.
25. Sağlık kuruluşlarında tekerlekli sandalye gibi araçlara kolaylıkla ulaşabilmekteyim.	Access to some facilities such as wheelchairs, walkers, etc. is provided in the health center.
26. Sağlık personeli tarafından ihtiyacımla ilgili tarafıma verilen eğitimleri anlayabiliyorum.	The educations that are given to me are such that I understand them.
27. İhtiyacımla ilgili bilgiler, sağlık personeli tarafından anlaşılır bir şekilde verilmektedir.	The information I need is expressed in simple language without the use of specialized words.
28. Sağlık çalışanlarıyla iletişimim yeterlidir.	Communication of health workers (doctor, nurse, midwife, etc.) with clients is appropriate.
29. Sağlık çalışanları, ihtiyacımla ilgili tarafıma verdikleri bilgiyi benim anlayıp anlamadığımdan emin olmaya çalışırlar.	Health workers try to make sure I fully understand the health information provided.
30. Sağlık kuruluşları sosyal ve ekonomik durumumu dikkate alarak gerekli düzenlemeleri yapmıştır.	My living conditions are taken into accounts, such as marital status, ability to pay, and cultural differences.

*: Red items are extracted items. Item 14 and item 16 (the white surfaces) were removed in the reliability analysis. Factor 1: (5,7,8,9,10,11,12,13) Acceptability (Kabul Edilebilirlik). Factor 2: (1,2,3,4): Accessibility (Erişilebilirlik). Factor 3: (20,21,30): Affordability (Ulaşılabilirlik). Factor 4: (15,23,24,25,26,27,28,29): Accommodation (Uyum ve Uygunluk).
